# The Maternal Nutritional Buffering Model: an evolutionary framework for pregnancy nutritional intervention

**DOI:** 10.1093/emph/eoz037

**Published:** 2020-01-21

**Authors:** Zaneta M Thayer, Julienne Rutherford, Christopher W Kuzawa

**Affiliations:** 1 Department of Anthropology, Dartmouth College, Hinman Box 6047, Hanover, NH 03755, USA; 2 Department of Women, Children and Family Health Science, University of Illinois Chicago, 845 S. Damen Ave., MC 802, Chicago, IL 60612, USA; 3 Department of Anthropology and Institute for Policy Research, Northwestern University, 1810 Hinman Ave, Evanston, IL 60208, USA

**Keywords:** pregnancy, supplementation, fetal programming, placenta, birth outcomes, DOHaD

## Abstract

Evidence that fetal nutrition influences adult health has heightened interest in nutritional interventions targeting pregnancy. However, as is true for other placental mammals, human females have evolved mechanisms that help buffer the fetus against short-term fluctuations in maternal diet and energy status. In this review, we first discuss the evolution of increasingly elaborate vertebrate strategies of buffering offspring from environmental fluctuations during development, including the important innovation of the eutherian placenta. We then present the Maternal Nutritional Buffering Model, which argues that, in contrast to many micronutrients that must be derived from dietary sources, the effects of short-term changes in maternal macronutrient intake during pregnancy, whether due to a deficit or supplementation, will be minimized by internal buffering mechanisms that work to ensure a stable supply of essential resources. In contrast to the minimal effects of brief macronutrient supplementation, there is growing evidence that sustained improvements in early life and adult pre-pregnancy nutrition could improve birth outcomes in offspring. Building on these and other observations, we propose that strategies to improve fetal macronutrient delivery will be most effective if they modify the pregnancy metabolism of mothers by targeting nutrition prior to conception and even during early development, as a complement to the conventional focus on bolstering macronutrient intake during pregnancy itself. Our model leads to the prediction that birth weight will be more strongly influenced by the mother’s chronic pre-pregnancy nutrition than by pregnancy diet, and highlights the need for policy solutions aimed at optimizing future, intergenerational health outcomes.

Lay summary: We propose that strategies to improve fetal macronutrient delivery will be most effective if they modify the pregnancy metabolism of mothers by targeting nutrition prior to conception and even during early development, as a complement to the conventional focus on bolstering macronutrient intake during pregnancy itself.

## INTRODUCTION

Work in the past three decades has highlighted the importance of prenatal nutrition as an influence on developmental and adult biology in offspring [1, 2]. The most common approach to human work in this area has linked lower birth weight (BW), which is used as a proxy for prenatal undernutrition or stress, to cardiovascular diseases and other adult chronic degenerative conditions [1, 3]. The long-term effects of early environments often involve modifications in developmental biology, which trace in part to epigenetic modifications that influence tissue and organ function via modified regulation of gene expression [4, 5].

Although developmental and epigenetic sensitivity persists across the life span, many systems have periods of heightened sensitivity during which exposures have especially robust and long-lasting effects. For mammals in particular, many of these ‘sensitive’ or ‘critical’ periods overlap with stages of direct resource transfer between mother and offspring across the placenta or through breast milk [6]. The model that has emerged from the developmental origins literature is one in which the mother’s nutrition and her exposure to environmental factors can have lingering effects on the function and health of offspring systems as they grow and develop [7].

From a public health and policy perspective, these findings have led to hopes that the burden of disease in future generations may be reduced by improving the health and nutritional status of pregnant women [8]. However, a predominant focus on retrospective studies, and on longitudinal cohort studies that collect observational data, means that few long-term studies have focused on developing maternal interventions to harness these periods of heightened developmental sensitivity to improve offspring health outcomes across the life course [9]. As such, it is presently less certain whether *enrichment* of a mother’s otherwise marginal nutrition, during early development, pregnancy or both, improves the intrauterine environment sufficient to yield long-term improvements in offspring health and human capital [10, 11].

A long-standing research tradition has, however, evaluated nutritional impacts on birth outcomes [12, 13], which provide a useful, albeit imprecise [14], proxy for maternal–fetal nutrient transfer. Micronutrient supplementations (e.g. vitamin B, folic acid, iron) have had some notable successes in improving birth outcomes, including by increasing BW and neonatal survival rates in populations with imbalanced or deficient diets [15]. In contrast, studies that provide pregnant women with supplements of macronutrients (i.e. fat, carbohydrates and protein) tend to find an improvement in maternal nutritional status and reduced risk of stillbirth and neonatal death, but relatively modest impacts on offspring BW. For instance, a study assessing the impacts of nutritional supplementation among 1296 pregnant women from rural villages in Burkina Faso found a modest, non-significant improvement in BW of 31 g among women in a mixed macro- and micronutrient supplementation group relative to controls provided with multiple micronutrient supplements [16]. These findings are consistent with the findings of a Cochrane Review evaluating the effects of this and 10 other balanced energy/protein supplementation trials in pregnancy among 5385 women that reported an average increase in offspring BW of only 41 g [17], or the equivalent of ∼1% of the average US BW of 3800 g. The study with the largest effect size, which found a 136 g increase in BW in the offspring of severely undernourished Gambian mothers supplemented with an energy dense biscuit, did not include a micronutrient control group, thus making it unclear the extent to which micronutrient versus macronutrient supplementation drove this increase in BW [18]. Thus, while supplementation-related improvements in perinatal survival are certainly important, the failure of macronutrient supplementations to substantively improve BW suggests that enthusiasm for harnessing the nutritional sensitivity of early development to improve the health of future generations has outpaced efforts to test and refine effective intervention approaches [11].

Here we develop the Maternal Nutritional Buffering Model, an evolutionary and physiological framework for stimulating new approaches to public health interventions aimed at improving fetal nutrition and downstream health outcomes [also see 11]. We situate our discussion of supplementation in the context of placental function, and the differential pathways and consequences for macronutrients and micronutrients. Our goal is not to comprehensively review the rationale for nutritional interventions, nor do we cover all previously tested or possible maternal intervention targets. We argue that the effects of macronutrient supplementation during pregnancy on offspring size, a proxy of fetal nutrient availability, are dampened by homeostatic mechanisms that evolved to insulate the fetus against transient shifts in nutrient availability, including short-term nutrient deficiencies experienced by a mammalian mother. We believe that the Maternal Nutritional Buffering Model can help explain several observations, including (i) the increased efficiency of micronutrient as opposed to macronutrient supplementations in pregnancy on BW, and (ii) the finding that longer-term macronutrient supplementations are more effective than those that occur only during pregnancy. We hope that this synthesis inspires novel approaches to improving long-term offspring health via early and sustained enrichment of maternal experience and diet.

## EVOLUTION OF MATERNAL BUFFERING

Consider supplementing the diet of a pregnant woman. In the simplest, hypothetical scenario, the resources consumed are directly transferred to the fetus, and thus, the intervention has maximal efficacy. In this type of direct connection between maternal exposure and fetal experience, fetal access to resources is entirely dependent on the mother’s current intake. In fact, such examples are relatively rare, and serendipity of this sort is particularly unlikely for resources required in large and constant quantities, such as macronutrients. As we review below, vertebrate evolution has been marked by increasingly elaborate maternal buffering strategies aimed at maintaining stability in the offspring’s rearing environment despite environmental fluctuations in factors such as temperature and nutrient availability.

### Maternal buffering from fish to mammals

Modern day fish are descendants of the earliest vertebrates, which evolved only limited capacities for parental buffering of offspring (Fig.�1). In early vertebrates and the majority of fish species today, the adult female’s body forms eggs wherein a flexible cell membrane encases a nucleus of DNA, along with a small cytoplasmic store of nutrients, hormones and other factors needed to sustain early embryonic development following fertilization [19]. With the exception of a few species of cartilaginous fish that are viviparous [20], eggs are often deposited into the aqueous environment in large mats, awaiting external fertilization by males who release sperm directly into the water. In many species, the vast swarm of offspring generated at fertilization are on their own, with few fish species feeding their young. Notably, some species have developed more advanced strategies of parental care and guard larva or eggs by either protecting their territory or by brooding fry in their mouths [21].

**Figure 1.**
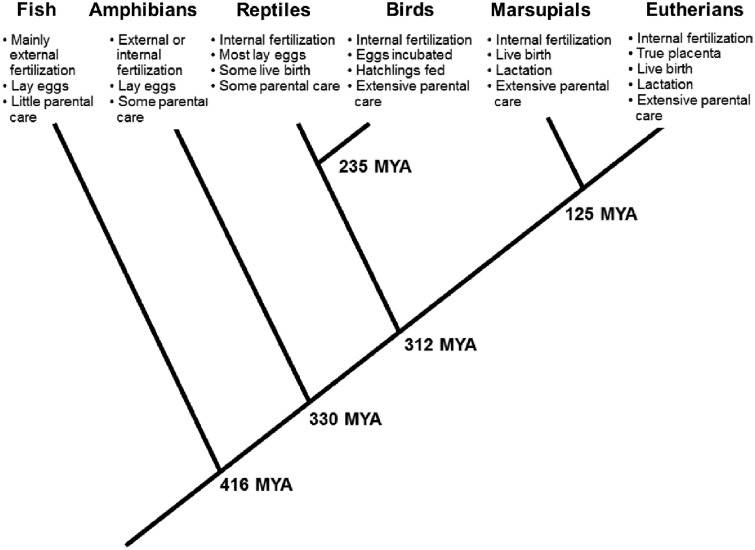
Evolutionary innovations in maternal buffering of offspring rearing environment. Dates (mya, million years ago) represent minimum fossil-based estimate for last common ancestor for each branching point [based on 126]. Giving birth to live young (viviparity) has evolved independently more than 100 times among marsupials, eutherian mammals, reptiles and even some species of fish, pointing to the evolutionary advantages of this strategy. Of the various reproductive strategies, the eutherian mammalian profile of internal fertilization, having a true placenta, and giving birth to live young that subsist on breast milk provides many opportunities for maternal biology to buffer environmental fluctuations and also to modify offspring development

Approximately 330 million years ago (mya), the lineage leading to modern amphibians diverged from their last common ancestor with modern fish. Some amphibians derived elaborate maternal investment strategies beyond egg laying. For instance, in some species of Caecilians (one of three forms of currently living amphibians), offspring eat the parents' nutrient-rich skin, which is rapidly regenerated [22]. However, with the exception of certain species that practice parental care in the form of egg attendance or tadpole transport and feeding [23], most amphibians simply lay eggs in water where they await external fertilization.

Between 330 and 310 mya the earliest amniotes evolved. These terrestrial species evolved more durable membrane-covered shells capable of surviving outside water. This group eventually gave rise to reptiles and birds in one lineage and mammals in a separate lineage. In all these species fertilization is internal, resulting in a more stable thermal, nutrient and hormonal environment during early embryonic growth. All birds and the majority of reptiles lay fertilized eggs, while non-monotreme mammals and some reptiles independently evolved the capacity for live birth. The precise temporal origin of placental mammals is unresolved, but has been placed within a broad range of ∼65 to 100 mya [24]. These early mammals were likely similar to modern marsupials. In marsupials, an internal placenta develops as a derivative of the yolk sac. This buffers the developing embryo from full immersion in the external environment for a brief time [25]. Marsupial offspring emerge from the maternal body during embryonic or very early fetal development and enter a pouch where mammary glands supply nutrients and hormones that support offspring growth [26]. This mammalian strategy of buffering was further augmented with the evolution of chorionic placentation in eutherian mammals, including humans, which allowed for extended periods of internal gestation. In these species a placenta, deriving from the fertilized egg, develops within the mother’s uterus and attaches to the uterine wall, creating a supply line between maternal circulation and the growing embryo.

### The innovation of the placenta

In placental mammals, the fetal compartment is embedded within the mother’s homeostatically regulated supply of circulating nutrients. Within this context, placentas are the interface between maternal and fetal biology, and they serve multiple roles: they facilitate the transport of essential resources like nutrients and gasses, they remove waste products generated through the fetoplacental unit’s metabolic activities, and they also help shield the fetus from exposure to potentially harmful compounds. Among placental mammals there is extensive variation in the gross morphological type of the placenta [27]. Most primates, including humans, have a hemochorial placenta, wherein maternal and fetal circulations are separated by 1–3 highly attenuated cell layers. As we will discuss further below, this helps facilitate nutrient transfer between the maternal and fetal compartments.

Nearly all of the maternal and fetal exchange takes place in the syncytiotrophoblast lining of the placental villi, as do the majority of placental endocrine and metabolic functions [28–30]. In addition to its role as a conduit for water, oxygen and carbon dioxide, the primary substrates transported and metabolized by the placenta are glucose, amino acids and fatty acids [31], all of which are essential for fetal development. Jansson and Powell have proposed that the placenta functions as a ‘nutrient sensor’ that is responsive to maternal nutrient supply and fetal nutrient demand [32]. In this view, the placenta senses the nutrient needs of the growing fetus and also the nutrient availability of the mother. Maternal nutrient supply drives the regulation of placental nutrient transport and allocation of nutrients to the fetus. Within this buffered context, the placenta has additional capacities to alter and mediate nutrient transport to the fetus, including the secretion of hormones into the mother’s circulation that modify maternal metabolism in ways that enhance nutrient delivery to the fetus, making it a key regulator of fetal growth and fetal developmental programming [33–35].

Simple diffusion of a molecule across the placenta can occur in both directions (i.e. towards the mother or towards the fetus), but the direction is dependent on a concentration or electrical gradient as well as structural factors such as the thickness of the vasculosyncytial membrane separating fetal and maternal circulations in human placentas [31, 36]. Fat-soluble molecules such as oxygen and carbon dioxide easily diffuse across the lipid-rich cell membrane and are more dependent on the concentration gradient between maternal and fetal circulations than on membrane area or thickness [31]. In contrast, water-soluble molecules like glucose and amino acids have limited capacity to diffuse passively across cell membranes [37]. Their passive transport is largely dependent on the concentration of the molecule in the maternal circulation as well as a greater amount of surface area of the vasculosyncytial membrane to ‘catch’ the molecule. The extensive microscopic surface area and the thin (1 and 2 �m) membrane of the fetomaternal interface thus helps maximize transport of these critical water-soluble molecules [31].

Active transport enhances the transfer of water-soluble molecules across lipid-rich membranes, and can move substrate against the maternal–fetal gradient. This is important when the goal is to have a consistently high substrate concentration in fetal circulation, such as with some amino acids, which are crucial building blocks used in the synthesis of proteins and peptide growth hormones [38]. Maternal conditions that relate to lower BW (e.g. high-altitude hypoxia) are associated with reduced amino acid transport function, while maternal conditions that relate to higher BW (e.g. gestational diabetes) increase amino acid transport function [38]. Glucose transport is also highly dependent on the density of transporter proteins within the membrane; low BW babies born to mothers living at high-altitude throughout pregnancy have placentas with reduced glucose transporter density [39]. This demonstrates the ability of the placenta to sense changes in the environment and functionally adapt in ways that influence fetal development.

## PATHWAYS LINKING MATERNAL AND FETAL ENVIRONMENTS

The above review highlights the diversity of evolved strategies that allow maternal biology to buffer the micro-environment of early offspring development. Although modern reptiles, birds and mammals are contemporaries and thus equally ‘evolved’, placental mammals have particularly refined strategies that allow maternal biology to buffer the fetus from environmental variability. This innovation of balancing continuous transport and buffering across the placenta allowed for longer periods of maternal-offspring physiological and metabolic exchange during early development than is generally seen in fish, amphibians, reptiles and birds. This basic understanding provides a starting point for considering two distinct pathways of intergenerational nutrient transfer—those buffered by maternal stores only (essential micronutrients) and those homeostatically regulated via stores and *de novo* synthesis (macronutrients)—that link maternal diet and metabolism with the intrauterine environment experienced by offspring (Table�1).

**Table 1.** Pathways linking maternal exposures to fetal exposures

	Pathway A	Pathway B
	Essential nutrient	Macronutrient
Examples	Vitamins (A, C, D, E, K and B complex), minerals (i.e. iron, copper, zinc, fluoride, selenium)	Carbohydrates (i.e. glucose), protein (amino acids), fatty acids
Effect on development	Co-factors in metabolic processes; deficit leads to developmental impairment	Energy substrate and building blocks of all cells and tissues; primary drivers of growth and metabolic programming
Quantity	Trace quantities are typically required for healthy development	Large quantities are required
Source	Diet, maternal stores	Diet, maternal stores and other metabolic precursors
Buffering capacity	Buffering of dietary deficits by maternal stores	Circulating levels are homeostatically maintained with input from diet, extensive bodily stores and metabolic interconversion between substrate types
Effect of nutritional intervention in pregnancy on fetus	If maternal stores depleted, can have direct/immediate effects	Dietary nutrients enter maternal metabolism which dampens direct effects on current pregnancy
Recommended intervention targets	Supplementing maternal intake during and prior to pregnancy	Supplementing during mother's own early development and across life course

### Pathway A. Essential nutrients—buffered by body stores only

Maternal biology is the sole source of nutrients and vitamins required for healthy fetal development. Some nutrients required in small quantities, in particular micronutrients such as vitamins and minerals, are classified as *essential* because they are not produced within the body and thus must be consumed preformed from dietary sources. Many micronutrients serve as co-factors in metabolic or enzymatic processes, and infants born with micronutrient deficiencies are at increased risk for adverse outcomes like low BW, neural tube defects and preterm delivery [40]. Some micronutrients are essential because they cannot be produced by organic chemistry (e.g. essential metals), while in other instances human ancestors lost the capacity to synthesize certain factors over the course of evolutionary history because they were required in trace quantities readily met by ancestral diets. As one well-known example of the latter type, humans’ inability to synthesize ascorbic acid (vitamin C) is thought to be due to the fact that humans evolved from fruit-eating primates, for whom vitamin C was easily acquired from the diet [41].

The body has some capacity to store most essential nutrients to help ensure that needs are met should intake fall below demand (Fig.�2A). When endogenous stores of essential nutrients are present, homeostatic regulation generally assures that circulating levels remain constant [42]. Looking across species that vary in habitual diets, the capacity to store a micronutrient varies substantially [42], perhaps pointing to the fact that evolutionary selection has tended to calibrate storage capacities to habitual patterns of availability and use. However, the storage capacity for some key micronutrients are modest compared to the increase in demands during pregnancy and lactation, when recommended intakes are thus generally increased [43, 44]. When diets are deficient in specific nutrients, this may limit a woman’s ability to meet these increased needs. For example, total iron requirements across pregnancy for a 55 kg woman are ∼1000 mg, which may exceed the quantity that can be absorbed from the diet during pregnancy. As a result, women need iron stores of at least 300 mg entering pregnancy to maintain optimal iron status [43].

**Figure 2.**
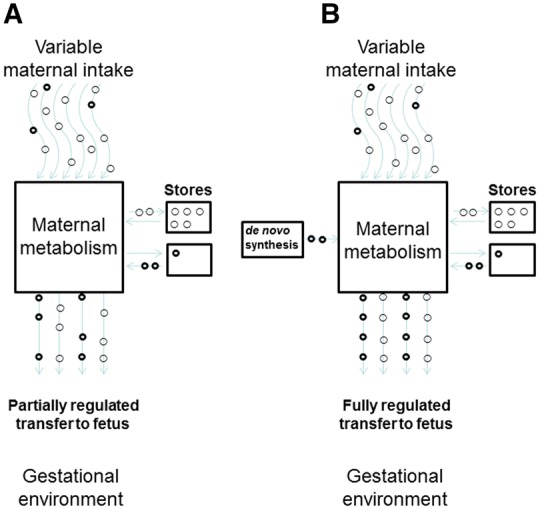
Pathways linking maternal intake of a nutrient or compound with fetal exposure to that compound. (A) Essential nutrient—a beneficial resource that the body is not capable of adequately synthesizing *de novo*. Delivery of adequate levels of the resource to the fetus is contingent upon dietary intake and the size of the mother’s bodily stores. (B) Major macronutrients—internal availability is homeostatically regulated by dietary intake, mobilizing tissue stores and through *de novo* synthesis from precursors. In light of these redundant sources, nutrient delivery to the fetus is often unrelated to the mother’s current dietary intake. Maternal regulatory set points that govern nutrient transfer to the fetus may be more effective targets for intervention

Vitamins are essential in that they must come preformed from the diet. Vitamin D is an exception in that it can be produced in variable amounts by the body in response to sun exposure, though vitamin D deficiency is common worldwide and dietary supplementation is recommended during pregnancy [45, 46]. Vitamins are transported across the vasculosyncytial membrane into fetal blood by various modes, depending on whether they are water- or fat-soluble. Water-soluble vitamins, which are stored in limited quantities, are transported across placental membranes into the fetal circulation via both diffusion and active transport [47]; active transport allows ready uptake by the fetal circulation. Fat-soluble vitamins are transported across the vasculosyncytial membrane, both via facilitated diffusion, a slower process than active transport (vitamins D, E and K), and by receptor-mediated diffusion (vitamin A) [48].

Given the importance of micronutrients for fetal development and the small size of maternal stores compared to reproductive needs, it is not surprising that maternal micronutrient supplementations during pregnancy often substantively improve maternal and child health outcomes [13, 49], although over-supplementation can be problematic [50, 51]. There is also evidence that micronutrient supplementations during pregnancy can affect offspring DNA methylation [52, 53]. For example, a study of perinatal micronutrient supplementation among marginally nourished Gambian women resulted in epigenetic modifications in offspring at 9 months of age in genes associated with resistance to infection and immune function, pointing to the likelihood that micronutrient supplementation could yield more durable benefits in other systems and outcomes [54]. In populations where low BW is a critical public health concern, micronutrient deficiencies are more likely than are abundances, and supplementation has been successful in increasing BW in some cases.

Pregnancy induces changes in maternal metabolism that increase the efficiency with which some essential nutrients are extracted from the diet and utilized, which can further augment the effectiveness of interventions [55]. For example, in rats vitamin B12 given during pregnancy is preferentially transferred to the fetoplacental unit in lieu of bolstering maternal stores, suggesting mechanisms to help ensure micronutrient supply to the developing fetus [56]. In humans the efficiency of absorption of some nutrients also increases in pregnancy [57, 58].

In summary, pregnancy often requires an increase in dietary consumption of micronutrients relative to the non-pregnant state [59, 60]. The mother’s ability to buffer fetal requirements of these scarce but critical resources therefore depends on several factors, including storage capacity within the human body, adequacy of maternal dietary intake and any cumulative deficits entering pregnancy, such as from prior pregnancies. As a result of these factors, in populations with unbalanced or marginal nutrition, micronutrient supplementation during pregnancy will often yield direct beneficial effects on offspring outcomes.

### Pathway B. Macronutrients homeostatically regulated via stores and *de novo* synthesis

The quantitatively most important resources required of offspring development are macronutrients: proteins, carbohydrates and fats. These provide the energy necessary for maintaining cellular function throughout the body and are the building blocks for gene products and structures like cellular membranes. Of the macronutrients, glucose is the primary energy substrate delivered between mother and fetus and is closely associated with BW variation. Clinical work has shown that maternal glucose levels directly determine fetal glucose supply [61, 62]. As discussed above, this is achieved by placental glucose transporters, the density of which are sensitive to maternal nutritional status [63]. Since fetal growth is insulin-driven, glucose transfer stimulates insulin production, and secondarily, fetal growth rate.

Given the pivotal role of circulating glucose to fetal growth rate, what determines maternal glucose level? Because energy substrates like glucose are required in large quantities to maintain constant functioning of every cell in the body, and since bodily requirements fluctuate across hours, weeks or even months, their availability is not relegated to chance (Fig.�2B). A mother’s diet is only one of several potential sources of glucose called upon to sustain maternal and fetal needs (Fig.�3). After a meal, foods are digested and broken down into constituent nutrients. Their presence stimulates the production of insulin, which initiates nutrient uptake by tissues and organs which use them for energy or to replenish carbohydrate, fat and protein stores. If dietary intake declines below use, stored substrates are mobilized, beginning with the body’s modest glycogen stores. As glycogen stores are depleted after several hours, the body turns to the more voluminous adipose tissue stores of triglycerides, which are broken down into glycerol and free fatty acids (FFAs). Glycerol enters the liver where it is converted into glucose via gluconeogenesis. The released FFAs are used as an alternative fuel source in liver, muscle and other tissues which induces insulin resistance in these tissues and thereby reduces their glucose uptake. This spares glucose for delivery to high priority non-insulin-dependent organs, including the brain, immune system and during pregnancy, the fetoplacental unit [64]. As a last defense, amino acids stored in muscle protein can also be mobilized and used as a gluconeogenic substrate, although preferential use of fats helps minimize the breakdown of lean tissues [65, 66].

**Figure 3.**
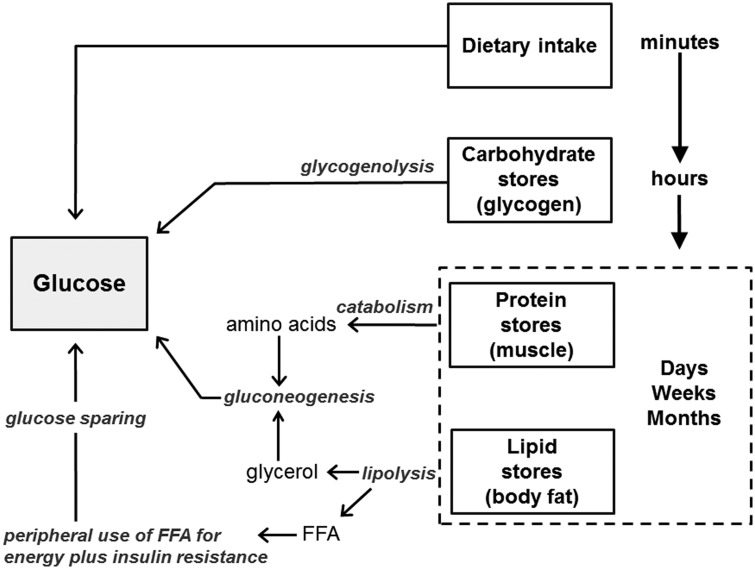
Redundant sources of glucose within the mother’s body. Glucose levels rise after consuming a meal. During a fast, circulating glucose is first maintained by mobilizing the body’s modest glycogen stores, which are sufficient to meet glucose needs for several hours. After glycogen stores are depleted, glucose is produced from mobilized amino acids (protein) and glycerol (fats). During prolonged fasts, peripheral tissues use fatty acids for energy and become insulin resistant, conserving glucose for obligate glucose-using functions and tissues. Although the brain generally only uses glucose, during starvation it may also use ketone bodies derived from mobilized fatty acids as an alternative fuel source. The shift to a predominant focus on fat metabolism with prolonged energy deficits reduces glucose requirements and thereby minimizes the need to catabolise protein in tissues and organs to provide substrate for glucose production

Although amino acids can be used as energy substrates they are also necessary for protein synthesis and accretion within the fetus [67], and maternal protein requirements are increased 30–50% during pregnancy [55]. Amino acids are mainly supplied by the diet, but may also be mobilized from muscle when dietary supplies are limited [55]. Critical amino acids are actively transported across the placenta [68]. Reduced or elevated transport of amino acids across the placenta, for instance due to maternal smoking or diabetes, respectively, is associated with intrauterine growth restriction or macrosomia in infants, demonstrating their importance as regulators of fetal growth [68].

FFAs are also delivered to offspring during gestation, either directly or as triglycerides (TG), which the placenta converts to FFA. They are used as energy sources, are essential to structures like cellular membranes, and late in gestation, are deposited in the human newborn’s unusually large store of body fat [69, 70]. Maternal fat accumulation predominates early in pregnancy, but maternal lipid metabolism switches to a catabolic state, resulting in increased TG and FFA concentrations in the last weeks of gestation [62, 71]. In cases of negative energy balance, adipose tissue lipolytic activity is enhanced, increasing FFA and glycerol, which are converted to ketone bodies and glucose, respectively, in the liver. These substrates easily cross the placenta and supply energy for the fetus [72].

Some FFAs have important structural or metabolic functions beyond serving as energy substrate. Although required in smaller quantities, some long-chain polyunsaturated fatty acids (LCPUFA) play an important role in fetal development, such as the well-known requirement of docosahexaenoic acid (DHA) for brain growth [73]. LCPUFAs are able to cross the placenta through active transport via transport proteins as well as through passive diffusion along the maternal–fetal concentration gradient [63]. There is evidence that fatty acids like DHA, which are challenging to synthesize *de novo* and are required in large quantities late in gestation, may be mobilized from maternal or fetal fat stores if dietary intake lags behind requirements [70].

During pregnancy, maternal metabolism is adjusted to prioritize glucose delivery to the fetus, which the fetoplacental unit itself helps orchestrate. The human placenta, with close comingling of fetal and maternal circulations, secretes more than 100 peptide and steroid hormones directly into the mother’s circulation, some of which alter maternal metabolism [74]. This includes placental lactogen, which induces insulin resistance in the mother’s peripheral tissues; the resultant decrease in maternal glucose uptake helps prioritize glucose delivery to the fetoplacental unit [75]. Such work shows that the placenta not only helps maintain a constant supply of nutrients through its role in regulating nutrient transport at the maternal–fetal interface, but also by directly manipulating maternal metabolism in ways that increase nutrient delivery from maternal peripheral tissues and organs [75].

To summarize, in contrast to the factors transported via Pathway type A above, macronutrients important for fetal growth are delivered to maternal peripheral tissues as a normal part of homeostatic metabolic regulation, and there are multiple pathways and backup sources for meeting supply-demand imbalances (Fig.�2B). The fetoplacental unit benefits from being embedded within this homeostatic system. The placenta can further stimulate nutrient availability to the fetus by altering expression of nutrient receptors, along with the effects of placentally derived hormones that are secreted into the maternal circulation and that actively shunt nutrients to the fetus. As a result of these various self-regulating systems, supplementing maternal diet during pregnancy, by itself, is unlikely to result in large effects on the fetus, since maternal metabolism, and not diet, is what shapes fetal macronutrient substrate availability.

## IMPLICATIONS OF THE MATERNAL NUTRITIONAL BUFFERING MODEL FOR NUTRITIONAL INTERVENTION DESIGN

Above, we note that modifying maternal exposure to essential micronutrients (Pathway A) can lead to relatively rapid and direct changes in fetal exposure, pointing to the utility of targeted nutritional interventions during pregnancy itself. In contrast, maternal delivery of the quantitatively more important macronutrients like glucose, fatty acids or amino acids—the primary determinants of fetal growth and metabolic programming—are not passive outcomes of what the mother eats that day but are homeostatically maintained within narrow limits by maternal metabolism and more locally by the placenta (Pathway B). Because maternal metabolism has multiple sources to meet substrate needs, the fetus is largely buffered against temporary shortfalls in maternal macronutrient intake. This buffering, however, appears to work both ways: the tendency of maternal metabolism to maintain a constant internal state also tempers the beneficial impact on the fetus of supplementing maternal diet during pregnancy.

Of course, macronutrient supplementation does benefit maternal nutritional status, health and offspring survival, and is thus well-justified [76, 77]. However, as discussed above, substantial increases in fetal growth rate and birth size have been challenging to achieve through such interventions. Rather than being delivered to the fetus, increases in maternal caloric intake during pregnancy appear to primarily allow increased maternal physical activity and energy expenditure, and perhaps also to enhance maternal fat deposition [78, 79].

The discussion of Pathway B illustrates that short-term supplements in essence ‘push’ against maternal buffering systems that are designed to absorb the impact of such short-term fluctuations—whether negative or positive—and protect metabolic set points and a stable internal milieu despite them. As we will argue, several independent lines of evidence converge on a common conclusion: policies must take a long-range view and also optimize early life nutritional conditions of the present generation if we hope to improve the nutrition that their offspring will receive *in utero*.

### Evidence that the mother’s pre-pregnancy and life course nutrition influence offspring nutrition and growth

Although nutrition during pregnancy itself is a weak predictor of offspring BW, there are clear links between maternal pre-pregnancy and life course nutrition and offspring BW. For instance, the well-known impact of the mother’s pre-pregnancy weight on fetal nutrition and growth [80] illustrates that fetal nutrition is influenced by the cumulative energy balance of the mother in the months and years prior to conception. Maternal stature-associated changes in pelvic dimensions are also particularly important predictors of fetal growth in humans, and link maternal developmental nutrition to offspring growth [81]. In some, but not all [82, 83], populations with nutritional growth stunting, the majority of adult height deficits compared to healthy growth references are present by 2–3 years of age and persist into adulthood [84, 85], reflecting deficits in length at birth combined with the impact of post-weaning nutritional stress and infectious morbidity on postnatal linear growth [86].

Consistent with these findings, the specific components of maternal size that correlate most strongly with offspring size suggest a lingering impact of early postnatal nutrition on offspring fetal growth [87]. For instance, in the Boyd Orr cohort in Britain leg length at 7 years of age was found to be a stronger predictor of offspring BW than was adult size [88], which was similar to findings in the 1958 British birth cohort [89]. In a study in the Philippines, leg length was a far stronger predictor than trunk length of offspring BW and placental weight [90]. Since childhood leg growth is the component of linear growth most sensitive to nutrition [91, 92], these findings suggest that nutrition in early life has lingering impacts on intrauterine nutrient transfer and fetal growth rate in offspring [6, 93].

The most widely documented evidence for intergenerational effects of nutrition and growth comes from multi-generational cohort studies that include information on BW across multiple generations [reviewed by 94]. These studies find robust relationships between maternal and offspring BW [95] which are strengthened after adjustment for gestational age, indicating that it is fetal growth rate, rather than differences in size due to prematurity, that tracks most closely across generations [96]. Moreover, these relationships are often independent of maternal adult stature, suggesting that there is a component of the intergenerational BW correlation that is not merely capturing an effect of birth size on later adult size [94].

While correlations between fetal growth rate in mother and offspring partly reflect an effect of shared genes, there is evidence for epigenetic and developmental contributions to these correlations, suggesting that early life nutrition can have lingering biological impacts on the next generation [97]. Studies generally report an excess in BW heritability through the matriline when compared to the patriline [98], showing that there are more than direct genetic effects underlying these relationships. Epigenetic contributions are a plausible explanation for this finding [98], and gain support from human studies. As one example, women whose mothers experienced the Dutch Famine while pregnant with them gave birth to offspring who were themselves slightly smaller [99] and who had reduced methylation near the *IGF2* gene [100], which in humans is an imprinted gene that effects metabolism and fetal growth [75].

### Phenotypic inertia: is maternal nutrient transfer a source of ecological information?

Given the exquisite mammalian capacity to buffer the fetus against changes in current maternal intake, how do we make sense of the fact that fetal growth is modified in response to maternal nutritional experience years or even decades in the past? One possibility is that the associations are simply driven by constraint, with nutritional deficiencies experienced by mothers adversely affecting their own development and that of their offspring [101]. However, a link between the mother’s growth rate and offspring BW is also predicted in light of the fact that they are both products of the mother’s expenditure of excess metabolic potential (‘productivity’), which is first used to support her own growth before being shunted in support of offspring growth in adulthood [see 93, 102]. In addition, multiple authors have speculated that some instances of nutrition-driven fetal developmental plasticity allow the fetus to prepare for conditions likely to be experienced after birth [6, 103, 104]. Some of the adjustments made by the nutritionally stressed fetus *in utero*, such as a tendency to deposit more abdominal body fat, and the reduced response of muscle to insulin, which spares glucose, could provide advantages if the postnatal environment is also nutritionally stressful [for review see 64]. Similarly, evidence reviewed above that offspring BW is sensitive to the mother’s own early life nutrition suggests a maternal capacity to recalibrate reproductive expenditure in response to early life nutritional cues [93, 105].

In contrast to micronutrients delivered via Pathway A, macronutrients that are both buffered via stores and also produced *de novo* via maternal metabolism (Pathway B) are relatively decoupled from current intake, and as discussed above, appear to reflect a mother's chronic, pre-pregnancy and developmental nutritional conditions [6, 105, 106]. Based on this, it has been hypothesized that the flow of macronutrients to the fetus provides a long-term average index of maternal nutritional experience. In an unpredictable environment this ‘backward looking’ form of adaptation, or *phenotypic inertia* [6], may provide a best guess about conditions that offspring are likely to experience in the future [107].

Although the pathways linking a mother’s own early life nutrition with her offspring’s fetal nutrition and growth are not well understood, one candidate mechanism for phenotypic inertia is the pre-placental nutritional reliance of the embryo on endometrial glands, which deliver resources to the developing embryo for the first 10–12 weeks of gestation, until the maternal circulatory supply to the placenta is more fully established [108]. Prior to nutrient delivery from the maternal blood supply, endometrial secretions supply carbohydrates, proteins and lipids to the embryo via communication with the intervillous space, which will eventually fill with maternal blood. Germinal epithelium from which mature endometrial glands form is already present during the mother’s own fetal life [109], implying that the qualities of the endometrium partly reflect the grandmother’s gestational nutritional conditions. Because the establishment of the placenta is part of a complex cascade of interactions between the invading trophoblast and the endometrium with which it comes into contact [74], characteristics of the endometrium prior to implantation could play a role in shaping placental function and thus nutrient transport for the duration of gestation. The ontogeny of the endometrial gland illustrates how factors that influence the gestational nutritional environment could have effects that cascade across multiple generations of offspring, thus highlighting the potential for multi-generational benefits of interventions that succeed in improving fetal nutrition and growth.

Regardless of the specific mechanisms involved, the concept of phenotypic inertia informs the Maternal Nutritional Buffering Model by implying that homeostatic systems that buffer fetal nutrition do so in part because natural selection has likely shaped maternal and placental physiology to provide integrative, and thus more reliable, information to guide offspring development. This suggests an additional reason why maternal biology might buffer the fetus against not only nutritional stress but also nutrition that is better than average: because an unusual improvement in nutrition is likely transient, it would be unwise to plan future expenditure to expect continued abundance. So long as there survival or other fitness costs associated with over-reaching one’s nutritional supply, the organism should ignore temporary, short-lived increases in maternal intake when setting developmental trajectories [6]. Because maternal biology and metabolic status develop in response to nutrition early in life, across the growing years, and in the adult years prior to conception, maternal biology embodies a cumulative record of a lifetime of past nutritional experiences and is thus a candidate source of historical cues of those experiences [6, 10, 110]. This model leads to the general hypothesis that measures of chronic, developmental nutritional sufficiency will be stronger predictors of offspring BW when compared to maternal macronutrient intake during pregnancy itself. Specific mechanistic predictions derived from the model are listed in Table�2.

**Table 2.** Directional predictions arising from the Maternal Nutritional Buffering Model

Maternal/placental biology	Predicted effect of a long-term supplementation intervention
Endometrial gland macronutrient content	Increased
Placental glucose transporters	Increased density
Placental amino acid transporters	Increased density
Microscopic placental surface area	Increased
Vasculosyncytial membrane thickness	Decreased
Placental weight relative to BW	Increased

According to the proposed model, we would predict that sufficient long-term nutritional conditions, which may be achieved with a long-term supplementation intervention, would be associated with changes in specific aspects of maternal and placental metabolic/nutrient biology.

## CONCLUSIONS

The Maternal Nutritional Buffering Model points to the need to tailor interventions based on the pathway through which a factor influences fetal development. In the case of resources derived strictly from the environment and which are partially buffered by maternal stores, such as essential vitamins and nutrients, supplementing women prior to and during pregnancy is likely to have substantial effects, especially when maternal nutrient status and dietary intake are marginal. This likely explains the relative success of many pregnancy micronutrient supplementations, such as folic acid, iron and vitamins B12, D and A [40, 60, 111].

In contrast to the above scenario, supplementing women with macronutrients during pregnancy alone is unlikely to achieve the full potential benefits associated with improving fetal nutrition because maternal metabolism has evolved mechanisms to buffer offspring from transient fluctuations in macronutrient intake. The evolutionary and physiologic background that we review helps explain the relatively modest effects of pregnancy macronutrient supplementation on birth outcomes [17, 76]. Because pregnancy supplementations emulate a short-term change in intake, they engage primarily with homeostatic buffering systems, which by their nature work to minimize the impacts of ecological variability on internal state. In contrast, longer-term changes in the mother’s experiences, starting with her own early development and continuing with weight gain and body composition during the pre-conceptional years, may be more effective at emulating stable changes, thus leading to a commitment to an increased nutritional plane in offspring [9]. In this sense, supplementations that target pregnancy itself can be viewed as emulating the wrong timescale of change, and thereby engaging with adaptive processes that maintain stability rather than change course.

Although available studies are few, there is evidence that sustained improvements in maternal nutrition can result in relatively large changes in offspring BW. For example, undernourished Guatemalan women who were provided a high quality protein–calorie supplement (*atole*) during one pregnancy had infants that were 124 g heavier than a control group, while women who were supplemented across one pregnancy and the lactation period of a preceding pregnancy had infants that were 150 g heavier. Women who were supplemented across two pregnancies and the intervening lactation period gave birth to infants that were 301 g heavier [112]. In a follow-up of the offspring and grandoffspring, daughters whose mothers were supplemented with *atole* as children gave birth to offspring who were 116 g heavier than mothers provided with the less nutritious supplement [113]. These findings support the idea that fetal growth tracks maternal nutritional history, and points to the need to consider nutritional interventions of young infants and children, as well as more sustained efforts across adulthood, as an integral component of any strategy to improve the nutritional experiences, birth outcomes and long-term cardiometabolic health of future generations [114].

The historical and intergenerational nature of prenatal nutrition underscores the need to promote policies focused on achieving long-range public health goals. Given the importance of nutrition for both maternal and offspring health, political will and financial commitments will be required to ensure adequate access to nutrition not only during pregnancy but also across the life course of multiple generations to improve population health [114]. Although we have emphasized the health benefits of improved fetal development, work that demonstrates the economic returns predicted by proxies of fetal gestational conditions have the potential to appeal to a broader array of policy makers and stakeholders [115, 116].

Our coverage in this review has by necessity been relatively selective, and we solely focused on maternal contributions to fetal nutrition and growth. Similar principles likely apply to infant metabolic programming via maternal nutrients and hormones in breast milk. Similar to fetal nutrition, breast milk composition responds directly to maternal intake of essential nutrients [117], but is unrelated to maternal macronutrient intake [118, 119]. Notably, there is some evidence for stronger links between macronutrient composition and the mother’s chronic or early life experience [120]. In addition, there is growing evidence from animal models that paternal nutrition can also have intergenerational influences on birth size and offspring health, likely through epigenetic modifications [121, 122]. Although studies investigating similar questions in humans remain scarce, there is tentative evidence for transgenerational effects of paternal and grandpaternal nutrition on offspring metabolism and disease risk [123, 124]. Thus paternal diet and life course experiences could have underappreciated effects on offspring development, and point to the need for future research to also explore the multi-generational benefits of paternal nutritional supplementation. Finally, our focus on understanding evolved, adaptive strategies for buffering against short-term nutritional deficiencies means that we do not address the evolutionarily recent phenomenon of chronic overnutrition. Obesity and the associated problem of the metabolic syndrome are associated with dysregulation of the mother’s homeostatic biology, which can lead to excess circulating concentrations of glucose that drive rapid fetal growth and postnatal weight gain [125]. This increasingly common intergenerational cause of offspring obesity risk once again speaks to the power of maternal metabolism as a primary driver of fetal growth—in this case represented by chronically elevated maternal glucose secondary not to diet per se, but to insulin resistance and other symptoms of the metabolic syndrome.

In sum, the Maternal Nutritional Buffering Model recognizes the evolution of distinct pathways linking different types of nutritional resources to the intrauterine environment experienced by the fetus. While essential micronutrients derived from the diet and from limited maternal reserves are good candidates for interventions during pregnancy, optimal improvements in delivery of homeostatically regulated resources, such as most macronutrients, may require long-term approaches that modify the mother’s own development and metabolism to emulate longer timescales of ecological change.
